# Chemical, biochemical, preclinical and clinical studies of *Ganoderma lucidum* polysaccharide as an approved drug for treating myopathy and other diseases in China

**DOI:** 10.1111/jcmm.13613

**Published:** 2018-04-24

**Authors:** Pengjiao Zeng, Zhihua Guo, Xuan Zeng, Cui Hao, Yiran Zhang, Meng Zhang, Yong Liu, Hui Li, Juan Li, Lijuan Zhang

**Affiliations:** ^1^ Systems Biology & Medical Center for Complex Disease Affiliated Hospital of Qingdao University Qingdao China; ^2^ School of Medicine and Pharmacy Ocean University of China Qingdao China

**Keywords:** cancer, clinical studies, *Ganoderma lucidum* polysaccharide, immunotherapeutic, myopathy

## Abstract

*Ganoderma lucidum* is an edible medicinal mushroom known as “Lingzhi” in China and “Reishi or Manetake” in Japan. It is a highly prized vitality‐enhancing herb for more than 2000 years. *G. lucidum* polysaccharide (GLPS) has been identified as one of the major bioactive components and developed into a drug named “Ji 731 Injection” in China since 1973. The large‐scale production of the drug began in 1985 and approved by the Chinese FDA as “Polysaccharidum of *G. lucidum* Karst Injection” (Ling Bao Duo Tang Zhu She Ye) in 2000, which is applied intramuscularly. After more than forty years of clinical use, its efficacy, safety and long‐term tolerability have been recognized by neurologists. It is one of a few non‐hormonal drugs used for treating refractory myopathy. It is also used for combination therapy, which reduces the amount of glucocorticoid required for myopathy patient who is in remission. In addition, it reduces adverse reactions and improves the quality of life for cancer patients during chemotherapy. We found 81 qualified chemical, biochemical, preclinical and clinical studies of GLPS both in English and in Chinese spanning from 1973 to 2017 by searching CNKI (China National Knowledge Infrastructure), Wanfang database and PubMed. The molecular mechanisms underlying GLPS's antioxidant, anti‐tumour, immune‐modulatory, hypoglycaemic, hypolipidaemic and other activities are discussed. Both preclinical and clinical studies are either deliberated or indexed in the current article. We aimed at providing a molecular picture as well as a clinical basis to comprehend GLPS as one of few polysaccharide‐based modern medicines with complicated chemical and pharmacological properties that prevent it from entering the world's market.

## 1 INTRODUCTION

**Figure 1:**
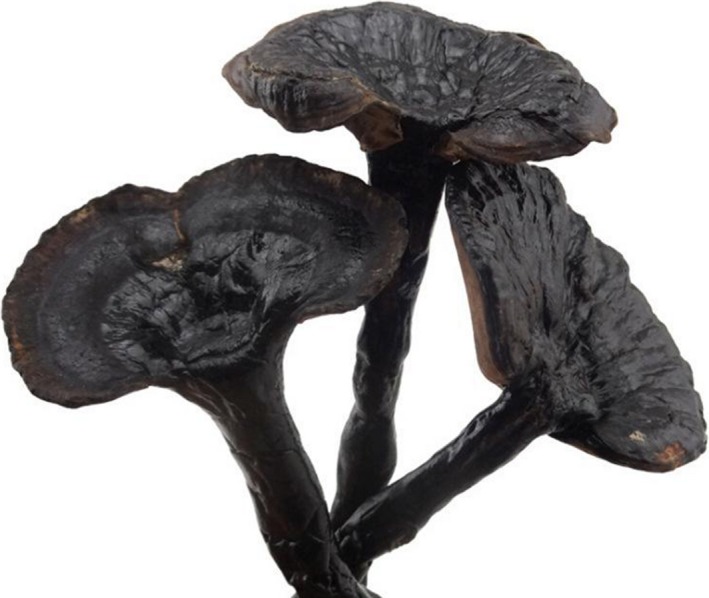
The picture of *Ganoderma lucidum*

*Ganoderma* is a genus of polypore mushrooms that grow on wood that include about 80 species. *Ganoderma lucidum*, an edible medicinal mushroom known as “Lingzhi” in China and “Reishi or Manetake” in Japan 1 (Figure 1), has been used for promoting health and for extending life in China and in other East Asia countries for more than 2000 years.^2^ There are multiple species of Lingzhi, scientifically known to be within the *Ganoderma lucidum* species complex.^3^ “Lingzhi” includes both *G. lucidum* and *G. sinense* as recorded in Chinese Pharmacopoeia of 2010.^4^, ^5^, ^6^

The fungi, mycelia and spores of *G. lucidum* contain about 400 different bioactive substances, including polysaccharides, triterpenoids, nucleotides, sterols, steroids, fatty acids, proteins/peptides and trace elements. Among them, *G. lucidum* polysaccharide (GLPS) has been identified as one of the major bioactive components, showing most physiological and health‐promoting effects acclaimed for *G. lucidum*, such as anti‐tumour^7^, ^8^, immune‐modulatory,^8^, ^9^ antioxidant,^10^, ^11^, ^12^, ^13^ hypoglycaemic^14^, ^15^ and other activities.

**Figure 2:**
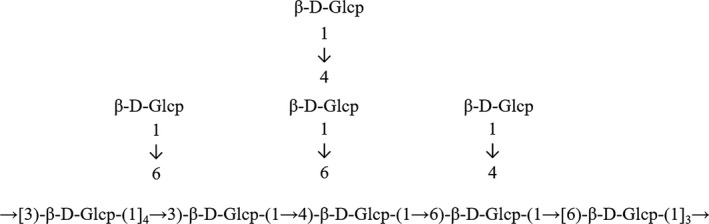
Structure of β‐glucan in *Ganoderma lucidum* polysaccharide, modified from Wang et al (2017)

*Ganoderma lucidum* polysaccharide is the major component by weight among all constituents in the spores of *G. lucidum*. Over 200 polysaccharides have been isolated and structurally articulated in the fruiting bodies, mycelia and spores of *G. lucidum*; however, modern analytical chemistry is still revealing new polysaccharides from *G. lucidum*.^12^ Glucose, mannose, galactose, xylose, fucose and arabinose have been identified in GLPS, and only β‐glucan, a pure glucose polymer, is believed to be one of the active ingredients in GLPS.^10^, ^16^ The β‐glucan structure in GLPS is shown in Figure 2.

*Ganoderma lucidum* is cultivated artificially on media of wood meal, rice bran and wood blocks in the last 10 years in China, Japan and the United States. However, these techniques do not guarantee a standardized yield as the compositions and structures of GLPS varied from batch to batch due to its non‐template biosynthesis. Baskar et al^17^, ^18^ reported that using statistical and evolutionary optimization methods and response surface methodology could enhance the production of *G. lucidum* MTCC 1039 in submerged culture. Thus far, how to standardize the biological activity and chemical structures of GLPS is still a pending issue.

So far, several *G. lucidum*‐based drugs are authorized by State Food and Drug Administration of China (SFDA) for clinical use. Table 1 shows the names, components, benefits, SFDA‐certified numbers and the number of manufacturers that produce the specific *G. lucidum*‐based drugs.

**Table 1:** Ganoderma *lucidum*‐based drugs approved by SFDA in China

Drug name	Components	Benefits	Drug numbers	No. of manufacturers
*Ganoderma* capsules	*Ganoderma* extracts	Insomnia, forgetfulness, physical weakness, neurasthenia	Z35020559 Z19993169 Z34020705 Z00812004 Z13021412	65
*Ganoderma* tablet	*Ganoderma* extracts	Insomnia, forgetfulness, physical weakness, neurasthenia	Z45022158 Z45020362 Z21021884 Z13021413 Z36020672	23
Compound *Ganoderma lucidum* granules	*Ganoderma* extracts	Chronic hepatitis	Z11021221 Z13020664 Z12020282 Z13020314 Z13020314	16
*Ganoderma lucidum* syrup	–	Palpitations, insomnia, loss of appetite, neurasthenia	Z53021201 Z32020893 Z45021449 Z36020812 Z32020921	12
*Ganoderma lucidum slices*	*Ganoderma lucidum* extract	Chronic hepatitis, neurasthenia, dizziness, insomnia, gastrointestinal ulcers, chronic bronchitis Coronary heart disease	Z20043150 Z20043199 Z20043459 Z34020483 Z20053282	7
*Ganoderma* longan wine	*Ganoderma lucidum* extract	Frail, post‐partum weakness, anaemia	Z45021420 Z45021095 Z36020578 Z45020259 Z36020662 Z45022143	6
*Ganoderma lucidum* spore capsules	*Ganoderma lucidum* spores	Heart and spleen deficiency, adjuvant therapy for cancer patients	B20040034 B20050008 B20040033 B20040035	4
*Ganoderma* granules	*Ganoderma lucidum* extract	Insomnia, forgetfulness, physical weakness, neurasthenia	Z19993223 Z35020571 Z45021930 Z45020361	4
*Ganoderma* oral liquid	–	Insomnia, forgetful, fatigue	Z20000010 Z20025750	2
Ginseng *Ganoderma* capsules	–	Physical weakness	Z20028001	1
*Ganoderma* dispersible tablets	*Ganoderma lucidum* paste	Insomnia, forgetfulness, physical weakness, neurasthenia, chronic bronchitis	Z20090141	1
*Ganoderma* dripping pills	*Ganoderma lucidum* extract	Insomnia, forgetfulness, physical weakness, neurasthenia	Z20090778	1
*Ganoderma lucidum Gynostemma* oral liquid	*Ganoderma lucidum* extract	Improve palpitations, insomnia	B20020517	1
Polysaccharidum of *Ganoderma lucidum* Karst Injection	*Ganoderma lucidum* Karst spore powder extract	Neurosis, polymyositis, dermatomyositis, atrophic myotonia Progressive muscular dystrophy	H20003510 H20003123	2
Polysaccharidum of *Ganoderma lucidum* Karst Injection	*Ganoderma lucidum* Karst spore powder extract	Neurosis, polymyositis, dermatomyositis, atrophic myotonia Progressive muscular dystrophy	H20051702	1

GLPS, *Ganoderma lucidum* polysaccharides.

**Figure 3:**
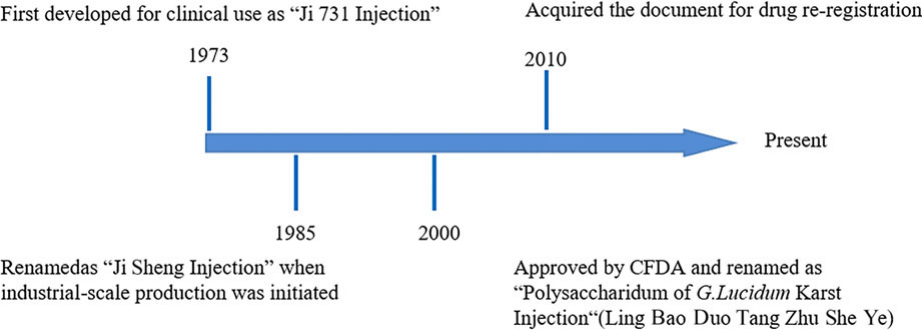
Timeline for developing Polysaccharidum of *Ganoderma lucidum* Karst Injection

A *G. lucidum* polysaccharide‐based product named “Ji 731 Injection” was used clinically for treating myopathy in China since 1973. The drug was independently developed by the Institute of Chinese Medical Sciences and its affiliated pharmaceutical factory (the Predecessor of Beijing Union Pharmaceutical Factory). Because of this, the institute won the 1978 National Award for Science and Technology. In 1985, it was renamed as “Ji Sheng Injection” before its large‐scale production. In 2000, State Food and Drug Administration of China (SFDA) approved and renamed the drug as “Polysaccharidum of *G. lucidum* Karst Injection” (Lin Bao Duo Tang Zhu She Ye) where the “Polysaccharidum of *G. lucidum*” is extracted from the spore of *G. lucidum*. The timeline for the drug development is shown in Figure 3.

Polysaccharidum of *G. lucidum* Karst Injection is applied intramuscularly. At present, this product is used for treating neurosis, polymyositis, dermatomyositis, atrophic myotonia and muscular dystrophy, and various diseases caused by a defective immune system. After more than forty years of clinical use, its efficacy, safety and long‐term tolerability have been recognized by neurologists. It is one of the few non‐hormonal drugs used for treating refractory myopathy. It is also used for combination therapy, which reduces the amount of glucocorticoid required for myopathy patient who is in remission. In addition, Polysaccharidum of *G. lucidum* Karst Injection reduces adverse reactions during chemotherapy and improves the quality of life of cancer patients significantly.

**Figure 4:**
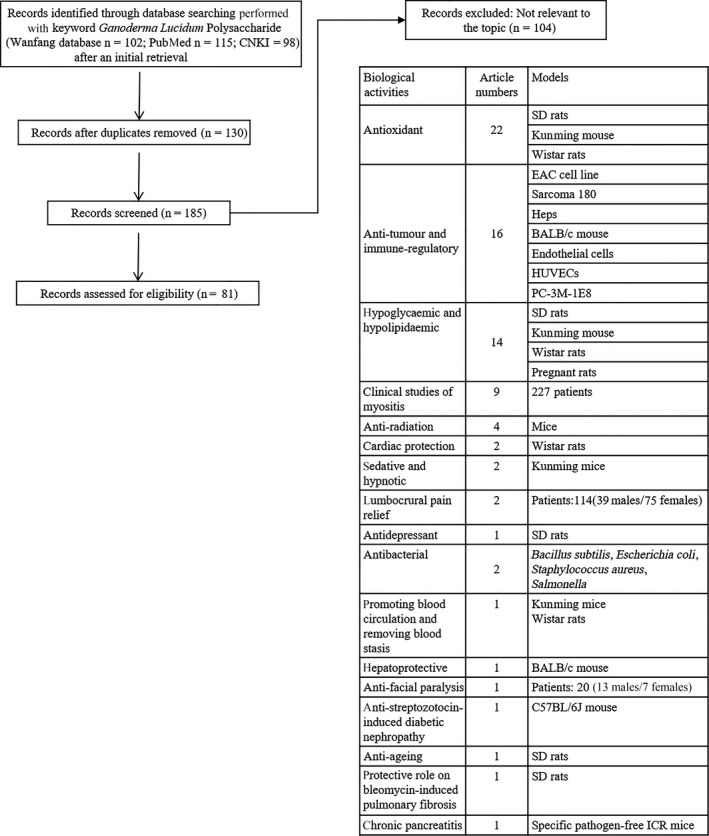
Flow chart of screened and analysed studies. Based on China National Knowledge Infrastructure (CNKI), Wanfang database and PubMed searches performed with keywords “*Ganoderma lucidum* polysaccharide,” “Ji 731,” “Ji Sheng,” “Polysaccharide of *Ganoderma lucidum* Karst Injection” and “Ling Bao Duo Tang Zhu She Ye,” we pulled out all of the publications. After reviewing the description about the *Ganoderma lucidum* polysaccharides (GLPS) used, the methods, data, results and conclusions, we found 81 qualified studies of GLPS both in English and in Chinese spanning from 1973 to 2017

Based on CNKI (China National Knowledge Infrastructure), Wanfang database, and PubMed searches performed with keywords “*Ganoderma lucidum* polysaccharide,” “Ji 731,” “Ji Sheng,” “Polysaccharide of *G. lucidum* Karst Injection” and “Ling Bao Duo Tang Zhu She Ye” both in English and in Chinese, we pulled out all of the publications. After reviewing the description about the GLPS used, the methods, data, results and conclusions, we found 81 qualified studies of GLPS both in English and in Chinese spanning from 1979 to 2017. A summary of the major biological activities of GLPS described in the qualified reports is shown in Figure 4, which demonstrates its immune‐regulatory, antioxidant, anti‐tumour, hypoglycaemic, hypolipidaemic, anti‐myositis, anti‐radiation, cardiac‐protecting, sedative, hypnotic, lumbocrural pain relief, antidepressant, antibacterial, blood circulation–promoting, blood stasis–removing, hepatoprotective, anti‐facial paralysis, anti‐streptozotocin‐induced diabetic nephropathy, anti‐ageing, anti‐bleomycin‐induced pulmonary fibrosis and anti‐chronic pancreatitis effects of GLPS.

## 2 IMMUNOMODULATORY AND ANTI‐TUMOUR ACTIVITIES OF GLPS

**Figure 5:**
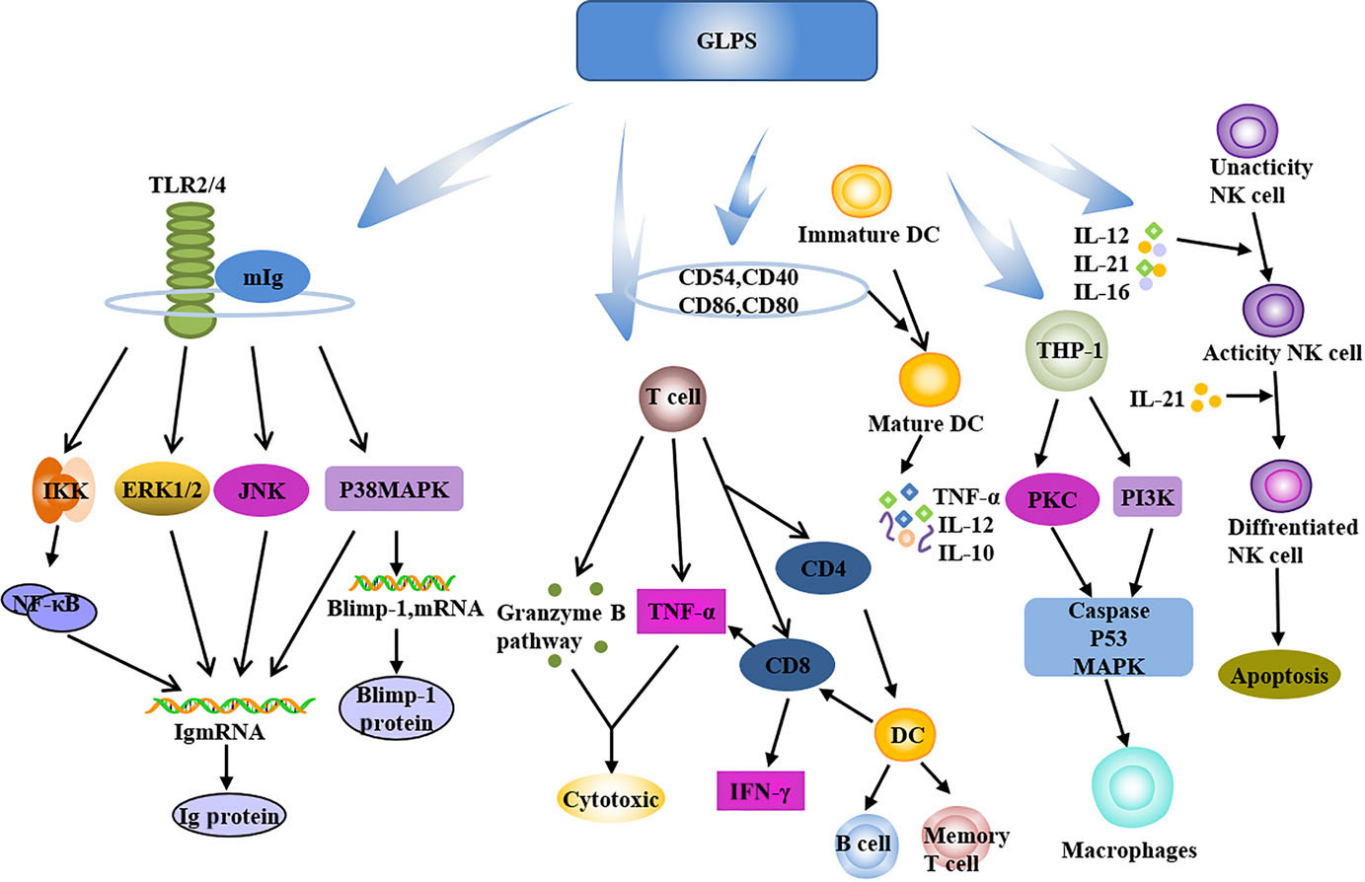
Immunomodulatory and anti‐tumour activities of *Ganoderma lucidum* polysaccharides (GLPS), modified from Xu et al (2011). Both innate and acquired immunities are enhanced by GLPS through activating T cells, B cells, NK cells, DC cells, antigen‐presenting cells and tissue macrophages accompanied by releasing a variety of chemokines, cytokines and growth factors

Multiple studies demonstrated that GLPS is a potent immunomodulator that exerts a significant and comprehensive impact on immune cells including B lymphocytes, T lymphocytes, NKs, macrophages and Dendritic cells (DCs). These immunomodulatory effects are likely to have been mediated by its complex multiple components and can be one of the underlying anti‐tumour mechanisms of GLPS to some extent. The potential effects of GLPS on immune cells are summarized in Figure 5.

Bao et al^19^ found that GLPS enhances T‐cell and B lymphocyte proliferation to some extent. Shao et al found that GLPS‐mediated B‐cell activation requires cell membrane Ig (mIg) and toll‐like receptor 4 (TLR4), which is also involved in GLPS‐mediated macrophage activation. GLPS enhances antibody secretion associated with Blimp‐1 mRNA induction. The biological function of Reishi‐F3 (a preparation of GLPS) is TLR4/TLR2‐dependent, and the interaction of Reishi‐F3 with TLR4/TLR2 is followed by the induction of the signal transduction of p38 mitogen‐activated protein kinase (p38 MAPK) in Blimp‐1 mRNA. At the same time, Reishi‐F3‐mediated Ig secretion is involved in the signalling of Extracellular signal‐regulated kinase, p38 MAPK, JNK and IκB kinase complexes. Cao et al^20^ found that GLPS can increase the expression of IFN‐γ mRNA and granzyme B protein expression. It can also promote the cytotoxicity of T lymphocytes induced by DC‐specific, and the mechanism of cytotoxicity is presumed to be mediated by IFN‐γ and granzyme‐mediated B pathway. GLPS promotes the release of TNF‐γ and IFN‐γ in T lymphocytes in a dose‐dependent manner.^21^ Zhang et al^22^ showed that GLPS is a new B cell–stimulating factor, B‐cell percentage and mouse spleen B lymphocyte activation increased by three‐ to fourfolds.

Lin et al^23^ showed that the treatment of DC with GLPS results in enhanced cell surface expression of CD80, CD86, CD83, CD40, CD54 and human leucocyte antigen‐DR. GLPS‐induced activation and maturation of human monocyte–derived DCs are mediated by NF‐κB and p38 MAPK pathways. GLPS can also stimulate monocyte‐derived DC maturation. GLPS and Granulocyte macrophage colony‐stimulating factor (GMCSF)/IL‐4 simultaneously induced THP‐1 cells to transform into a typical DC form, whereas GLPS alone induced proliferation in THP‐1 and U937 cells alone. Furthermore, the transformation of THP‐1, DCs results in a significant increase in the expression of HLA‐DR, CD40, CD80, and CD86 and in similar antigen‐uptake capability. Thus, GLPS can effectively promote the activation and maturation of immature DCs, preferring the Th1 response.^24^

GLPS from *G. lucidum* spores can increase the volume of macrophages and their ability to devour milk beads.^25^ The pharmacological inhibitor assay showed that the ability of GLPS to enhance phagocytosis and chemotactic neutrophil function is mediated by PI3K, p38 MAPK, Src tyrosine kinase and Protein kinase C.^26^ Chien et al^27^ showed that the fucose‐containing glycoprotein fraction (F3) isolated from the water‐soluble extract of *G. lucidum* stimulated CD56+ NK activity in umbilical cord blood. After the F3 treatment, NK cell–mediated cytotoxicity is found to be significantly enhanced. The polysaccharide component of the branched (1→6)‐β‐D‐glucan moiety, named *G. lucidum*‐G (GS‐G) has been reported to exhibit anti‐tumour activity and to activate NK cells.^28^

## 3 BIOLOGICAL ACTIVITIES OF GLPS

### 3.1 Antioxidant activities of GLPS

#### 3.1.1 Scavenging free radicals by GLPS

Free radicals refer to atoms, molecules or ions that have unpaired electrons. These unpaired electrons make free radicals highly chemically reactive towards other substances or even towards themselves. Reactive oxidative species (ROS) (such as $O_{2}^{-}$,**˙**OH, H_2_O_2,_ R**˙**, RO**˙**, ROO**˙)** and Reactive nitrogen species (such as NO**˙**, ONOO^−^) are biologically relevant free radicals. Under normal circumstances, human body constantly produces and removes free radicals to maintain a dynamic balance. A moderate number of free radicals in the body play an active role in cell division, differentiation, growth and elimination of bacteria and parasites. If free radicals are excessively produced or insufficiently removed, they can cause host protein denaturation, enzyme inactivation, nucleic acid metabolic abnormalities and the formation of lipid peroxides. The free radicals also cause the formation of protein aggregates through cross‐linking, which damages the structure and function of a cell, promotes senility and creates various pathological states in human and animals.

In general, all known polysaccharides have antioxidant activity that can be measured by mixing with a free radical compound DPPH. Several studies showed that GLPS can directly scavenge oxygen free radicals in a GLPS concentration–dependent manner.^29^, ^30^ Malondialdehyde (MDA) is a lipid peroxide formed when oxygen free radicals attack polyunsaturated fatty acids. Pretreatment of GLPS significantly reduced levels of ROS and MDA produced by myocardial cells; thus, GLPS plays a direct role in reducing myocardial oxidative stress.^31^ Similarly, when PC12 cells are pre‐treated for 2 hours with GLPS, it antagonizes the oxidative stress induced by Amyloid β(25‐35) (Aβ_25‐35_) that elevates the level of intracellular ROS.^32^

#### 3.1.2 Inhibiting oxidative stress in different animal models

In reality, by catching ROS, GLPS blocks or slows oxidative stress in different animal models (Table 2). In a rat model of Aβ‐induced Alzheimer's disease, GLPS inhibits the increased level of MDA both in serum and in hippocampus and protects rat neurons from oxidative stress.^33^, ^34^ GLPS also reduces lipid peroxidation in skeletal muscle of exhausted mice and ultimately protects the liver and skeletal muscle from oxidative stress.^16^ In a rabbit model of liver ischemia‐reperfusion injury, GLPS reduces the level of MDA significantly in liver and prevents liver from damage caused by oxygen free radical–mediated lipid peroxidation.^31^

**Table 2:** Antioxidant effect of GLPS in different mouse and rat models

Models	MDA/(μmol/L)			SOD/(U/L)		GSH‐Px/(U/L)		References
	Groups	Concentration	*P*‐value	Concentration	*P*‐value	Concentration	*P*‐value	
SD rats cardiomyocytes	Normal control	0.27 ± 0.032	–	40.85 ± 2.98	–	–	–	88
	H_2_O_2_	1.01 ± 0.078	<.01	16.77 ± 2.01	<.01	–	–	
	PSG‐1‐L (25 mg/mL)	0.59 ± 0.068	<.01	26.98 ± 2.21	<.01	–	–	
	PSG‐1‐M (50 mg/mL)	0.43 ± 0.045	<.01	32.69 ± 2.89	<.01	–	–	
	PSG‐1‐H (100 mg/mL)	0.32 ± 0.043	<.01	38.98 ± 3.09	<.01	–	–	
SD rats (10/group)	Normal control	3.61 ± 1.27	–	149.10 ± 5.93	–	–	–	33
	AD model	20.53 ± 3.34	<.01	84.79 ± 7.7	<.01	–	–	
	GLPS treatment	8.67 ± 2.67	<.01	128.75 ± 11.67	<.01	–	–	
SD rats (12/group)	Normal control	66.11 ± 7.64	–	113.14 ± 18.61	–	–	–	34
	AD model	14.18 ± 1.99	–	257.79 ± 38. 55	–	–	–	
	GLPS treatment	21.95 ± 5.79	<.05	135.76 ± 7. 97	<.05	–	–	
Kunming mouse (10/group)	Normal control (myocardium)	–	–	84.30 ± 11.3	–	5.71 ± 0.77	–	34
	GLPS (myocardium)	–	–	87.30 ± 10.2	–	7.11 ± 1.32	<.01	
	Normal control (skeletal muscle)	–	–	43.2 ± 7.3	–	30.7 ± 3.1	–	
	GLPS (skeletal muscle)	–	–	54.2 ± 7.7	<.01	29.3 ± 1.98	–	
Rabbits (10/group)	Normal control	3.12 ± 0.43	–	326.59 ± 28.63	–	–	–	31
	Normal saline	6.03 ± 0.29	<.05	223.32 ± 35.50	<.05	–	–	
	GLPS	4.86 ± 0.33	<.05	278.15 ± 24.41	<.05	–	–	
SD rats (10/group)	Normal control	2.71 ± 0.87	–	249.56 ± 16.38	–	23.76 ± 4.56	–	38
	DM	7.95 ± 1.49	<.01	131.06 ± 9.40	<.01	13.61 ± 2.53	<.01	
	GLPS‐L (200 mg/kg)	7.95 ± 1.21	–	131.94 ± 11.43	–	13.79 ± 4.47	–	
	GLPS‐M (400 mg/kg)	4.11 ± 0.69	<.01	178.15 ± 5.53	<.01	19.46 ± 3.65	<.05	
	GLPS‐H (800 mg/kg)	2.79 ± 0.49	<.01	187.66 ± 6.59	<.01	23.32 ± 2.54	<.05	
Mouse (10/group)	Ovarian cancer	–	–	261.21 ± 13.01	–	36.20 ± 6.20	–	40
	GLPS‐L (50 mg/kg)	–	–	304.11 ± 13.13	–	44.82 ± 5.62	–	
	GLPS‐M (100 mg/kg)	–	–	315.84 ± 11.09	–	45.60 ± 7.72	–	
	GLPS‐H (200 mg/kg)	–	–	330.83 ± 12.17	<.05	50.88 ± 15.42	<.05	
Wistar rats (10/group)	Normal control	205.71 ± 18.94	–	28.75 ± 1.83	–	39.75 ± 2.17	–	41
	Gastric cancer	137.89 ± 11.27	<.01	58.05 ± 4.01	<.01	61.52 ± 4.31	<.01	
	GLPS‐L (400 mL/kg)	189.33 ± 16.08	<.01	49.05 ± 3.28	<.01	61.52 ± 4.31	<.01	
	GLPS‐H (800 mL/kg)	216.48 ± 18.49	<.01	32.17 ± 2.17	<.01	45.06 ± 2.22	<.01	
SD rats (10/group)	Normal control	2.82 ± 0.99	–	246.59 ± 16.18	–	23.72 ± 4.76	–	42
	DM	7.98 ± 1.52	<.01	132.05 ± 9.10	<.01	13.52 ± 2.55	<.01	
	GLPS‐L (200 mg/kg)	7.98 ± 1.15	<.01	132.91 ± 11.33	<.01	13.70 ± 4.41	<.01	
	GLPS‐M (400 mg/kg)	4.06 ± 0.73	<.01	176.16 ± 5.41	<.01	19.42 ± 3.62	<.01	
	GLPS‐H (800 mg/kg)	2.82 ± 0.55	<.01	189.68 ± 6.55	<.01	23.16 ± 2.41	<.01	
Wistar rats (6/group)	Normal control	–	–	9.61 ± 0.49	–	11.15 ± 0.95	–	43
	DM	–	–	4.53 ± 0.07	<.01	5.76 ± 0.43	<.01	
	GLPS‐L (60 mL/kg)	–	–	5.07 ± 0.12	<.01	7.39 ± 0.29	<.01	
	GLPS‐M (80 mL/kg)	–	–	6.82 ± 0.54	<.01	7.39 ± 0.29	<.01	
	GLPS‐H (120 mL/kg)	–	–	8.67 ± 0.42	<.01	9.26 ± 0.28	<.01	

Models	Catalase/(U/L)			ROS		References
	Groups	Concentration	*P*‐value	Concentration	*P*‐value	
SD rats	Normal control	–	–	15.11 ± 0.95	–	88
	H_2_O_2_	–	–	62.54 ± 3.35	<.01	
	PSG‐1‐L (25 mg/mL)	–	–	26.09 ± 2.01	<.01	
	PSG‐1‐M (50 mg/mL)	–	–	19.38 ± 1.83	<.01	
	PSG‐1‐H (100 mg/mL)	–	–	16.52 ± 1.21	<.01	
SD rats (10/group)	Normal control	–	–	–	–	33
	AD model	–	–	–	–	
	GLPS	–	–	–	–	
SD rats (12/group)	Normal control	–	–	–	–	34
	AD model	–	–	–	–	
	GLPS treatment	–	–	–	–	
Kunming mouse (10/group)	Normal control (myocardium)	–	–	–	–	16
	GLPS (myocardium)	–	–	–	–	
	Normal control (skeletal muscle)	–	–	–	–	
	GLPS (skeletal muscle)	–	–	–	–	
Rabbits (10/group)	Normal control	–	–	–	–	31
	Normal saline	–	–	–	–	
	GLPS	–	–	–	–	
SD rats (10/group)	Normal control	25.34 ± 6.61	–	–	–	38
	DM	12.89 ± 1.69	<.01	–	–	
	GLPS‐L (200 mg/kg)	14.45 ± 2.68	–	–	–	
	GLPS‐M (400 mg/kg)	19.68 ± 3.72	<.01	–	–	
	GLPS‐H (800 mg/kg)	21.45 ± 3.21	<.01	–	–	
Mouse (10/group)	Ovarian cancer	7.57 ± 1.16	–	–	–	40
	GLPS‐L (50 mg/kg)	8.32 ± 0.89	–	–	–	
	GLPS‐M (100 mg/kg)	8.57 ± 0.76	–	–	–	
	GLPS‐H (200 mg/kg)	8.57 ± 0.76	<.01	–	–	
Wistar rats (10/group)	Normal control	–	–	–	–	41
	Gastric cancer	–	–	–	–	
	GLPS‐L (400 mL/kg)	–	–	–	–	
	GLPS‐H (800 mL/kg)	–	–	–	–	
SD rats (10/group)	Normal control	25.88 ± 6.62	–	–	–	42
	DM	12.98 ± 1.95	<.01	–	–	
	GLPS‐L (200 mg/kg)	14.37 ± 2.79	<.01	–	–	
	GLPS‐M (400 mg/kg)	19.79 ± 3.75	<.05	–	–	
	GLPS‐H (800 mg/kg)	21.47 ± 3.33	<.01	–	–	
Wistar rats (6/group)	Normal control	96.38 ± 6.29	–	–	–	43
	DM	53.86 ± 3.21	<.01	–	–	
	GLPS‐L (60 mL/kg)	70.65 ± 9.06	<.05	–	–	
	GLPS‐M (80 mL/kg)	78.86 ± 6.02	<.01	–	–	
	GLPS‐H (120 mL/kg)	88.12 ± 6.73	<.01	–	–	

DM, Diabetes mellitus; GSH‐Px, Glutathione peroxidase; GLPS, *Ganoderma lucidum* polysaccharides; ROS, Reactive oxidative species; SOD, Superoxide dismutase; MDA, Malondialdehyde.

All animals generate multiple antioxidant enzymes, such as Glutathione peroxidase (GSH‐Px), Superoxide dismutase (SOD) and Catalase (CAT). GLPS not only increases the expression of GSH‐Px, SOD and CAT that directly scavenges free radicals but also decreases the expression level of the enzymes that produce $O_{2}^{-}$, including NOX, XO and NOS. In fact, NOX is the main producer of ROS in vascular endothelial cells.^35^, ^36^, ^37^

As shown in Table 2, GLPS increases the activities of skeletal muscle SOD and myocardial GSH‐Px in mice and protects liver and skeletal muscle from damage in exhausted mice.^16^ Moreover, GLPS enhances the activity of GSH‐Px, SOD and CAT in serum and pancreas.^38^, ^39^ In an Alzheimer's disease model, GLPS stimulates the activity of SOD in serum and hippocampus in addition to accelerate the process of eliminating free radicals in the body.^35^ GLPS also increases the serum level of SOD, GSH‐Px and CAT in a mouse model of ovarian orthotopic tumour, indicating that GLPS enhances its sensitivity to chemotherapeutic drugs.^40^ In the rabbit model of liver ischemia‐reperfusion injury, GLPS significantly enhances the activity of SOD in liver of rabbit.^31^ GLPS‐treated rats have increased levels of serum and gastric tissue SOD, CAT and GSH‐Px compared to that of control in a dose‐dependent manner.^41^ GLPS at >400 mg/kg significantly enhances the GSH‐Px, CAT and SOD activities in the heart of type II diabetic rat and decreases myocardial MDA level at the same time.^42^ GLPS increases SOD, GSH‐Px and CAT levels in STZ‐diabetic rats as well.^43^

### 3.2 Anti‐tumour and immune‐regulatory activities of GLPS

Since 1970s, the anti‐tumour effect has been demonstrated for GLPS. Joseph et al^44^ found that GLPS at 100 mg/kg showed 80.8% inhibitory ratio of Ehrlich's ascites carcinoma (EAC) tumour cells (Table 3). Pang et al^45^ found that GLPS at 100 and 300 mg/kg exhibits stronger growth inhibition against S_180_, Hepatoma solidity cell and EAC tumour cells. GLPS almost has no adverse reactions to human body, and this advantage is not possessed in many tumour chemotherapy drugs and other immune promoter. When combined with chemo‐ or radiation therapy, GLPS can improve the healthy state of cancer patients and strengthen anti‐cancer effect of chemo‐ or radiation therapy, which makes GLPS an excellent adjuvant therapeutic drug for cancer patients.

**Table 3:** Anti‐tumour and immune‐regulatory effects of GLPS in both cell‐ and animal‐based models

Models	Inhibitory ratio/%		Other test indicators					References
	Groups							
Swiss albino mouse EAC	Normal saline	–	–					44
	Cyclophosphamide	71.8						
	GLPS‐L (25 mg/mL)	64.3						
	GLPS‐M (50 mg/mL)	73.4						
	GLPS‐H (100 mg/mL)	80.8						
ICR species mouse	Normal saline	–	–					45
1.S_180_	Cyclophosphamide	63.15						
	GLPS‐L (33.3 mg/kg)	23.73						
	GLPS‐M (100 mg/kg)	31.97						
	GLPS‐H (300 mg/kg)	38.56						
2.EAC	Normal saline	–						
	Cyclophosphamide	63.22						
	GLPS‐L (33.3 mg/kg)	19.66						
	GLPS‐M (100 mg/kg)	31.58						
	GLPS‐H (300 mg/kg)	35.89						
3. Heps	Normal saline	–						
	Cyclophosphamide	63.55						
	GLPS‐L (33.3 mg/kg)	24.08						
	GLPS‐M (100 mg/kg)	31.05						
	GLPS‐H (300 mg/kg)	35.73						
BALB/c mouse (10/group)			**NK cytotoxic activity/%**	**Phagocytosis ratio/%**	**IL‐2 (pg/mL)**	**IFN‐γ (pg/mL)**	**TNF‐α (pg/mL)**	46
	Normal control	–	35.6 ± 2.3	100.1 ± 11.0	–	–	–	
	Normal saline	–	8.3 ± 1.7	48.2 ± 5.0	–	11.73 ± 3.53	–	
	Cyclophosphamide	81	0.6 ± 0.5	–	–	–	–	
	GLPS‐L (50 mg/kg)	30.7	13.8 ± 1.0	75.0 ± 4.0	–	13.24 ± 3.48	–	
	GLPS‐M (100 mg/kg)	49.1	21.4 ± 2.9	134.1 ± 7.8	–	20.13 ± 2.93	20.62 ± 16.8	
	GLPS‐H (200 mg/kg)	59.9	30.3 ± 1.1	141.6 ± 27.8	3.4 ± 2.46	67.42 ± 5.47	–	
BALB/c mouse HL‐60 S_180_ L929 cells	Normal saline	–	**Apoptosis ratio/%**			***P*‐value**		48
	Cy (cyclophosphamide)	78.02	7.44 ± 1.07			–		
	GLPS‐L (50 μg/mL)	27.70	18.81 ± 0.93			<.01		
	GLPS‐M (100 μg/mL)	55.83	20.98 ± 1.57			<.01		
	GLPS‐H (200 μg/mL)	66.70	23.00 ± 0.56			<.01		
Chick chorioallantoic membrane PC‐3M‐1E8			**Angiogenesis inhibitory ratio/%**			**Adhesion of PC‐3M‐1E8 to laminin inhibitory ratio/%**		50
	Normal saline	–	–			–		
	GLPS‐L (0.2 μg)	–	14.8			23.89		
	GLPS‐M (1 μg)	–	28.1			35.4		
	GLPS‐H (5 μg)	–	46			39.82		
BALB/c mouse S_180_	Normal saline	–	–					51
	GLPS‐L (1 μg/mL)	35.2						
	GLPS‐M (10 μg/mL)	45.25						
	GLPS‐H (100 μg/mL)	61.88						
HUVECs PC‐23M	Normal saline	–	**Adhesion activity of PC‐23M inhibitory ratio/%**					52
	GLPS‐L (1 mg/mL)	–	43.90					
	GLPS‐M (10 mg/mL	–	41.46					
	GLPS‐H (100 mg/mL	–	58.54					
Endothelial cells	Normal saline	–	**Adhesion activity inhibitory ratio/%**			**Migration inhibitory ratio/%**		53
	GLPS‐L (50 μg/mL)	–	43.94			42.37		
	GLPS‐M (100 μg/mL)	–	42.83			44.86		
	GLPS‐H (200 μg/mL)	–	59.12			59.43		
	Cy (cyclophosphamide)	78.02	–					
	GLPS‐L (50 μg/mL)	27.7						
	GLPS‐M (100 μg/mL)	55.83						
	GLPS‐H (200 μg/mL)	66.7						

EAC, Ehrlich's ascites carcinoma; GLPS, *Ganoderma lucidum* polysaccharides; Heps, Hepatoma solidity cell.

#### 3.2.1 Inhibiting tumour cell proliferation

Studies showed that GLPS does not kill cancer cells directly in vitro,^46^, ^47^, ^48^ but it inhibits cancer cell proliferation, such as S_180_, PG and HL‐60 cells, in vitro, when GLPS‐treated animal serum is used in cell culture. It is proposed that one or more endogenous active substances are induced by GLPS, which are responsible for inhibiting cancer cell growth in vitro.

#### 3.2.2 Inducing apoptosis of cancer cells

Shang et al^49^ demonstrated that SeGLPS‐2B‐1, a Se‐containing polysaccharide purified and characterized from the Se‐enriched *G. lucidum*, induces MCF‐7 cell (human breast adenocarcinoma cell line) apoptosis via a mitochondrial pathway.

#### 3.2.3 Inhibiting tumour angiogenesis

Endothelial cell proliferation is one of the key steps in angiogenesis, which is essential for tumour growth. Thus, inhibition of vascular endothelial cell proliferation can inhibit tumour growth. As shown in Table 3, Zhang et al^50^ demonstrated that GLPS could significantly inhibit the angiogenesis in Chick embryo chorioallantoic membrane (CAM; at the administered dosages (0.2~5 g CAM); GLPS also remarkably inhibits with the adhesion of PC‐3M‐1E8 to laminin in a dose‐dependent manner at the concentrations of 0.33~3 g/L. Cao et al^51^ showed that the proliferation of HUVECs is inhibited by GLPS in a dose‐dependent fashion, and GLPS treatment of HUVECs could induce cell apoptosis directly. Human lung carcinoma cells when exposed to high dose of GLPS in hypoxia for 18 hours result in a decrease in VEGF secretion. They concluded that GLPS may directly inhibit vascular endothelial cell proliferation or indirectly decrease growth factor expression of the cancer cells.

#### 3.2.4 Inhibiting tumour metastasis

Li et al and Liang et al^52^, ^53^ reported that GLPS has no cytotoxicity towards PC‐3M in vitro, but GLPS could inhibit tumour cell adhesion and migration through the endothelium.

#### 3.2.5 Regulating the immune system

Studies showed that GLPS could enhance host immune function, activate the immune response and thereby inhibit tumour growth. As shown in Table 3, Wang et al found that the GLPS has no cytotoxicity towards S_180_ and PG cells in vitro. However, serum from GLPS‐treated S_180_‐bearing mice significantly inhibited S_180_ and PG cell proliferation in vitro. Moreover, GLPS promotes the splenic lymphocyte proliferation induced by ConA or LPS and enhances the cytotoxic activity of NK cells. The levels of serum IL‐2, IFN‐γ, TNF‐α and nitric oxide are also increased after GLPS treatment.^46^

### 3.3 Hypoglycaemic and hypolipidaemic activities of GLPS

As shown in Table 4, GLPS has both hypoglycaemic and hypolipidaemic effects, which are evidenced by the changed values of Fasting blood glucose (FBG), TC, TG, High‐density lipoprotein‐associated cholesterol (HDL‐C) and LDL‐C. Luo et al^54^ found that at gavage dose of 100 mg/kg or more, GLPS can reduce blood sugar levels in hyperglycaemia mice, and at gavage dose of 200 mg/kg, GLPS reduces blood sugar levels in normal mice. In addition, GLPS stimulates insulin secretion significantly in hyperglycaemic mice. In another study, Luo et al demonstrated that at gavage dose of 100 mg/kg, GLPS could significantly decrease the levels of serum TC, TG and LDL‐C. At gavage dose of 200 mg/kg, GLPS decreases the levels of serum TG and LDL‐C and increases the level of serum HDL‐C.^55^

**Table 4:** Hypoglycaemic and hypolipidaemic effect of GLPS

Models	FBG/(mmol/L)			TC/(mmol/L)		TG/(mmol/L)		References
	Groups	Concentration	*P*‐value	Concentration	*P*‐value	Concentration	*P*‐value	
Wistar rats (8/group)	Normal control	4.90 ± 0.70	–	0.78 ± 0.06	–	0.35 ± 0.08	–	62
	DM	16.93 ± 2.42	<.01	0.81 ± 0.07	–	0.51 ± 0.11	<.05	
	DM+GLPS	11.80 ± 1.71	<.01	0.74 ± 0.08	<.05	0.46 ± 0.05	<.05	
	DS (DM + sporting)	12.30 ± 2.10	<.01	0.75 ± 0.06	<.05	0.44 ± 0.06	<.05	
	DS+GLPS	9.18 ± 1.28	<.01	0.72 ± 0.04	<.01	0.40 ± 0.10	<.05	
Wistar rats (8/group)	Normal control	4.7 ± 0.2	–	2.46 ± 0.23	–	0.61 ± 0.04	–	61
	High‐fat/fructose diet	4.8 ± 0.2	–	2.55 ± 0.15	–	0.65 ± 0.07	–	
	DM	16.8 ± 0.7	–	3.78 ± 0.91	–	1.10 ± 0.05	–	
	PSG‐1	12.4 ± 1.0	<.01	2.59 ± 0.12	<.05	0.89 ± 0.08	<.05	
	Cyclosporin A	17.1 ± 1.4	–	2.80 ± 0.22	–	1.14 ± 0.04	–	
	N‐acetyl‐L‐cysteine	15.6 ± 1.2	–	2.78 ± 0.10	–	1.09 ± 0.13	–	
SD rats (10/group)	Normal control	2.99 ± 0.47	–	1.90 ± 0.12	<.01	0.84 ± 0.11	<.05	59
	DM	12.93 ± 1.91	–	2.87 ± 0.18	–	2.56 ± 0.73	–	
	GLPS‐L (100 mg/mL)	7.64 ± 0.94	<.01	2.38 ± 0.24	<.01	2.04 ± 0.18	<.01	
	GLPS‐M (200 mg/mL)	7.47 ± 0.87	<.01	2.29 ± 0.21	<.01	1.77 ± 0.27	<.01	
	GLPS‐H (400 mg/mL)	5.51 ± 0.77	<.01	2.15 ± 0.13	<.01	1.01 ± 0.19	<.01	
	Metformin	4.63 ± 0.80	<.01	2.08 ± 0.12	<.01	1.62 ± 0.12	<.01	
SD rats (10/group)	Normal control	–	–	1.35 ± 0.21	–	0.42 ± 0.23	–	64
	Hyperlipidaemia	–	–	9.13 ± 2.17	<.01	1.19 ± 0.21	<.01	
	GLPS‐L (200 mg/kg)	–	–	5.52 ± 1.29	<.01	0.83 ± 0.22	<.01	
	GLPS‐M (400 mg/kg)	–	–	6.23 ± 1.75	<.01	0.82 ± 0.22	<.01	
	GLPS‐H (800 mg/kg)	–	–	5.85 ± 1.62	<.01	0.80 ± 0.26	<.01	
SD rats (10/group)	Normal control	4.46 ± 0.44	–	1.49 ± 0.28	–	1.32 ± 0.24	–	63
	DM	22.60 ± 2.46	–	1.68 ± 0.20	–	1.60 ± 0.46	–	
	GLPS‐L (100 mg/kg)	12.04 ± 1.21	<.05	1.32 ± 0.24	<.05	1.04 ± 0.21	<.05	
	GLPS‐M (200 mg/kg)	9.82 ± 1.31	<.05	1.02 ± 0.20	<.05	0.92 ± 0.18	<.05	
	GLPS‐H (400 mg/kg)	8.69 ± 1.25	<.01	0.89 ± 0.19	<.01	0.85 ± 0.15	<.01	
SD rats (10/group)	Hyperlipidaemia	–	–	9.13 ± 2.17	–	1.19 ± 0.21	–	61
	GLPS‐L (100 mg/kg)	–	–	5.52 ± 1.29	<.01	0.83 ± 0.17	<.01	
	GLPS‐M (200 mg/kg)	–	–	6.23 ± 1.75	<.01	0.82 ± 0.22	<.01	
	GLPS‐H (400 mg/kg)	–	–	5.85 ± 1.62	<.01	0.80 ± 0.26	<.01	
SD rats (10/group)	Normal control	5.00 ± 0.00	–	2.01 ± 0.29	–	1.13 ± 0.16	–	60
	DM	25.00 ± 3.92	<.05	4.38 ± 0.34	<.05	1.74 ± 0.18	<.05	
	Metformin	13.53 ± 1.82	<.05	2.68 ± 0.36	<.05	1.38 ± 0.09	<.05	
	GLPS	16.61 ± 2.26	<.05	3.57 ± 0.64	<.05	1.54 ± 0.12	<.05	
Pregnant rats (8/group)	Normal control	5.6 ± 1.5	<.01	–	–	–	–	57
	DM	21.3 ± 1.9	–	–	–	–	–	
	GLPS	8.7 ± 1.6	<.01	–	–	–	–	
Kunming species mouse (8/group)	Normal control	5.98 ± 0.27	<.05	–	–	–	–	89
	DM	32.53 ± 0.37	–	–	–	–	–	
	GLPS‐L (100 mg/kg)	31.97 ± 0.22	–	–	–	–	–	
	GLPS‐M (200 mg/kg)	20.01 ± 0.14	<.05	–	–	–	–	
	GLPS‐H (400 mg/kg)	17.96 ± 0.49	<.05	–	–	–	–	
SD rats (16/group)	Normal control	4.38 ± 0.40	–	–	–	–	–	56
	DM	22.81 ± 1.41	–	–	–	–	–	
	GLPS‐L (0.1 g/kg)	11.33 ± 0.40	<.05	–	–	–	–	
	GLPS‐M (0.2 g/kg)	9.56 ± 0.23	<.05	–	–	–	–	
	GLPS‐H (0.3 g/kg)	7.23 ± 0.36	<.05	–	–	–	–	
Kunming species mouse (10/group)	DM	37.01 ± 7.80	–	–	–	–	–	54
	GLPS‐L (100 mg/kg)	22.40 ± 6.01	<.01	–	–	–	–	
	GLPS‐M (200 mg/kg)	23.87 ± 7.77	<.01	–	–	–	–	
	GLPS‐H (400 mg/kg)	23.82 ± 6.43	<.01	–	–	–	–	
Wistar rats (6/group)	Normal control	5.71 ± 0.7	–	–	–	–	–	43
	DM	22.14 ± 1.91	<.01	–	–	–	–	
	GLPS‐L (60 mg/kg)	17.32 ± 0.98	<.01	–	–	–	–	
	GLPS‐M (120 mg/kg)	14.38 ± 1.23	<.01	–	–	–	–	
	GLPS‐H (180 mg/kg)	8.43 ± 0.72	<.01	–	–	–	–	

GLPS, *Ganoderma lucidum* polysaccharides; TC, triglycerides; TG, total cholesterol; FBG, fasting blood glucose.

Zhang et al^56^ established an alloxan‐induced diabetic mouse model. Their data showed that GLPS decreases blood glucose level, increases insulin secretion, recovers the B cells and improves the liver glucokinase activity in the mouse model. Chen et al^47^, ^48^, ^49^ published three studies related to hypoglycaemic and hypolipidaemic effects of GLPS. They found that when STZ‐induced diabetic mice were treated with GLPS, levels of serum FBG, TC, TG and LDL‐C are decreased (*P* < .05) and levels of serum insulin and HDL‐C are significantly increased (*P* < .01). Zhao et al^50^ divided alloxan‐induced diabetic mice into normal saline, GLPS low‐dosage (GLPS‐L), GLPS middle‐dosage (GLPS‐M) and GLPS high‐dosage (GLPS‐H) groups and measured blood glucose levels after 0, 1, 2 and 4 hours. They found that all doses reduce blood glucose levels at all conditions tested with statistical significance (*P* < .05). Moreover, Shan et al^57^ found that GLPS could decrease blood glucose level and increase serum insulin level in gestational diabetic rats (*P* < .01). Decreased level of serum insulin and increased level of blood glucose are observed in the plasmas of untreated diabetic control rats. GLPS treatment increases serum insulin level and reduced blood glucose levels in STZ diabetic rats significantly and dose‐dependently.^43^ Huang et al^58^, ^59^ found that the high dose of GLPS is able to lower the levels of blood glucose, TC and TG and increase the level of HDL‐C in diabetic rats. GLPS exerts noticeable hypoglycaemic and hypolipidaemic effects (*P* < .05) in diabetic rats, and all the beneficial effects are better than those of Cyclosporine A and N‐acetyl‐L‐cysteamine (NAC).^60^, ^61^ Gong et al^62^ also found that combined sporting with GLPS could lower the levels of blood glucose and lipids in diabetic mice.

## 4 CLINICAL STUDIES OF GLPS ON MYOPATHY

As shown in Table 5, after treating with “Ji 731 Injection” for patients with mild, moderate and severe atrophic rhinitis, the effective rates are 80%, 67% and 33%, respectively.^63^ Subsequently, Fu et al^64^ reported that the *G. Lucidium* Karst extract has the ability to treat atrophic myotonia with 50% efficacy. Wang et al^65^, ^66^, ^67^ demonstrated that “Ji Sheng Injection” could be used to treat various myopathy diseases and the diseases of the nervous system including demyelinate diseases. Acupoint injection of *G. lucidum* Karst Injection had 75% significant efficiency of neurodermatitis.^68^ When acupoint injection of the Polysaccharidum of *G. lucidum* Karst Injection is combined with spleen‐arousing decoction, the efficacy ratio of treating low muscle strength in children was up to 90%.^69^

**Table 5:** Effect of Polysaccharidum of *G. lucidum* Karst Injection

Indications	Total number of cases (male/female)	Treatment group	Control	Effective rate (treatment/control)	References
Low muscle strength	51(35/16)	30, Polysaccharidum of *G. lucidum* Karst Injection	21, Current therapy	90%/47.6%	69
Neurodermatitis	64(24/40)	Acupoint injection of Ji Sheng	–	75%	68
Duchenne muscular dystrophy	17(14/3)	Ji Sheng Injection	–	64.71%	65
Atrophic myotonia	10	Ji Sheng Injection	–	50%	
Polymyositis and dermatomyositis	30(10/20)	Ji Sheng Injection	–	47.1%	
Hypothyroidism myopathy	1	Ji Sheng Injection +oral thyroid hormone tablets	–	Completely cured	
Myasthenia gravis	2	Ineffective when used alone and when shared with neostigmine enhance the efficacy			
Multiple sclerosis	15(6/9)	40% complete remission, 13.33% significant and 26.67% invalid			
JE vaccine injections after encephalomyelitis	1	Progress was not ideal, but therapeutic effect was better than any previously accepted			
Chronic multiple polyradiculoneuritis	4(3/1)	Ji Sheng Injection	–	50% completely cured	
Visceral ADHD	–	–	–	Completely cured	
Concomitant in medical illness	–	–	–	Completely cured	
Paralysis after cerebral arteriosclerosis and stroke	Enhance memory, improve their mood, and a variety of recovery after stroke has a certain effect				
Visceral ADHD (attention deficit/hyperactivity disorder)	2	Complete remission in a relatively short period of time, without recurrence			66
Atrophic myotonia	10(8/2)	50% effective, 20% increased and 30% improved			64
Atrophic rhinitis	20(3/17)	Significant efficiency: mild (80%), moderate (67%) and severe (33%)			63

## 5 OTHER PRECLINICAL AND CLINICAL STUDIES ON GLPS

As shown in Table 6, Polysaccharidum of *G. lucidum* Karst Injection has a curative effect on depression in a combination therapy.^70^ When combined with glucocorticoids, Polysaccharidum of *G. lucidum* Karst Injection is effective in treating facial paralysis in children.^71^ Zhang et al^72^, ^73^ conducted experimental studies on isolated heart preservation with Ji Sheng Injection and found that it can improve long‐term preservation of the isolated arrested rat hearts. Acupoint injection of Ji Sheng Injection could treat lumbar hyperplasia and lumbocrural pain with improved efficacy.^74^, ^75^ When intravenously injected at 80 mg/kg to rats, GLPS could prolong sleeping time and improve the sleeping quality.^76^, ^77^

**Table 6:** Other biological activities of GLPS

Function	Models	Groups		Test indicator	References
Facial paralysis	Patients: 20 (13 males/7 females)	10, Ling Bao Duo Tang Injection		After combination, glucocorticoid dosage reduced, reduced fast, course of treatment shorted and completely healed	71
		+ prednisone		Forehead symptom disappeared, mouth askew and other conditions improved significantly compared with before treatment	
		10, prednisone + VB_1_, VB_12_		In muscle function after four weeks of combination, there are very significant differences in the muscle function (*P* < .01)	
Lumbar hyperplasia	180 patients	90, acupoint injection of Ji Sheng		Efficacy: 98.9%	74
		30, Ji Sheng Injection		73.4%	
		60, electrotherapy		96.7%	
Lumbocrural pain	114 (39 males/75 females)	58(18/40), Ji Sheng + VB_12_		79.3%	75
		56(21/35), Stauntonia + VB_12_		64.3%, (*P* > .05)	
Depression	SD rats (8/group)	Normal control, depression		Level of 5‐HT, NE and DA increased than depression group (*P* < .05)	70
		Normal saline, GLPS		After treatment of GLPS 28 days, level of 5‐HT, NE and DA were equal to normal (*P* > .05)	
Cardiac protection	Wistar rats (10/group)	Modified Euro‐Collins solution (mEC)		Cardiac function and coronary flow were significantly better than mEC (*P* < .01)	72
		mEC + Ji Sheng Injection		The content of water and Malondialdehyde, in myocardium, was lower than mEC (*P* < .05)	
				Activity of LDH and CK was lower than mEC (*P* < .01), and SOD was higher (*P* < .05)	
Heart protection	Kunming mice (10/group)	Ji Sheng Injection‐L (400 mg/L)		Cardiac function and coronary flow were significantly better in three groups of Ji Sheng than mEC, especially in Ji Sheng‐H	73
		Ji Sheng Injection‐M (800 mg/L)		The content of water in myocardium in Ji Sheng‐M/H was lower than mEC (*P* < .01)	
		Ji Sheng Injection‐H (1600 mg/L)		Resurrection of heart rate was higher than mEC (*P* < .01)	
Sedative and hypnotic	Kunming mice (10/group)	Normal control, sodium pentobarbital + Ji Sheng Injection, Valium, Ji Sheng Injection		Extend sleeping time when administered along with sodium pentobarbital and sodium pentobarbital in a dose‐dependent manner	77
				Drug independence for Ji Sheng, but for Valium	
				Abnormal locomotor activity is significantly decreased	
Prolonged sleeping time	SD rats	Prolong sleeping time and improve sleeping quality			76
Anti‐radiation	Mice		Leucocyte/×10^9^/L	SOD/(U/mL)	79
		Normal control	12.28 ± 2.51	411.07 ± 9.18	
		Radiation	1.41 ± 0.26	357.39 ± 13.52	
		GLPS	1.92 ± 0.55	397.16 ± 5.92	
Anti‐radiation	Mouse		Leucocyte/×10^9^/L		78
		Normal control	12.08 ± 1.15		
		Radiation	2.14 ± 1.83		
		GLPS	2.37 ± 1.48		
Antibacterial	Inhibited Bacillus subtilis, Escherichia coli, Staphylococcus aureus and Salmonella				80
Antibacterial	Inhibited Bacillus subtilis, Escherichia coli, Staphylococcus aureus and Salmonella				81
Antibacterial	A strong inhibiting effect on Erwinia carotovora and a weak inhibiting effect on Penicillium digitatum				82
Blood stasis	Kunming mice	Prolonged clotting time and reduced serum TG levels in hyperlipidaemia mice inhibited thrombus formation in vitro			86
	Wistar rats				
Hepatoprotective	BALB/c mouse	Significantly mitigated hepatic tumefaction and decreased both ALT release and NO production			84
STZ‐induced diabetic nephropathy	C57BL/6J mouse	Reduced the serum Cr and BUN levels and oxidative stress			85
Anti‐skin ageing	SD rats (10/group)	Enhanced both hydroxyproline and SOD contents in a GLPS dose‐dependent manner			83
Protective roles on bleomycin‐induced pulmonary fibrosis	SD rats	Increased levels of glutathione, glutathione peroxidase, catalase and superoxide dismutase and decreased contents of malondialdehyde and hydroxyproline in the lung			90
Chronic pancreatitis	ICR mice	Alleviated the pancreatitis in mice through decreasing lipase, AMS, IFN‐γ and TNF‐α levels as well as increasing SOD and total antioxidant activity			91

GLPS, *Ganoderma lucidum* polysaccharides; SOD, Superoxide dismutase.

The level and activity of SOD is one of the important indicators to measure the body recover after irradiation. Studies demonstrated that GLPS could increase the level of SOD and the numbers of leucocytes.^78^, ^79^ Three independent studies have investigated the antibacterial activity of GLPS.^80^, ^81^, ^82^ Lin observed the anti‐skin ageing function of GLPS. In this model, GLPS increases both hydroxyproline and SOD contents in a concentration‐dependent manner, indicating that GLPS slows the skin ageing process.^83^ Furthermore, studies showed that GLPS is hepatoprotective^84^ in that it corrects the metabolic abnormalities of diabetic mice and prevents or delays the progression of diabetic renal complications.^85^ Finally, GLPS promotes blood circulation and removes blood stasis.^86^

## 6 FUTURE PERSPECTIVES

The data collected from published studies and presented in Figures 1, 2, 3, 4, 5 and Tables 1, 2, 3, 4, 5, 6 in the current manuscript provide solid evidence that GLPS has multiple biological functions and should be considered as an effective modern medicine. Both advantages and disadvantages of GLPS as a drug rely on their complicated molecular structures and multiple biological functions. When compared with small molecule‐ and/or protein‐based drugs, GLPS has broader spectrum of therapeutic properties but lacks specific molecular targets. Interestingly, it was reported that among 656 US FDA‐approved drugs tested, each drug hits more than 7 targets in the 73 total targets tested.^87^ In addition, many drugs are less effective to the previously known targets compared to off‐targets. Therefore, multiple or unknown biological targets in vivo might be a common but not a particular problem for GLPS.

In reality, multiple ingredients with multiple beneficial effects are essence of traditional Chinese medicines, which explains why GLPS is approved only by SFDA so far. However, there are multiple issues needed to be addressed before GLPS is accepted by governments and clinicians worldwide, such as how to comprehend the pharmacodynamics of GLPS, how to standardize the quality of GLPS and how to perform reliable pharmacokinetic studies of GLPS. Perhaps, the efficacy but not the homogeneity of polysaccharide‐based drugs, such as GLPS, should be emphasized by the drug regulators worldwide in the near future.

## CONFLICTS OF INTEREST

The authors declare no conflict of interest.
